# LMPID: A manually curated database of linear motifs mediating protein–protein interactions

**DOI:** 10.1093/database/bav014

**Published:** 2015-03-16

**Authors:** Debasree Sarkar, Tanmoy Jana, Sudipto Saha

**Affiliations:** Bioinformatics Centre, Bose Institute, Kolkata, India

## Abstract

Linear motifs (LMs), used by a subset of all protein–protein interactions (PPIs), bind to globular receptors or domains and play an important role in signaling networks. LMPID (Linear Motif mediated Protein Interaction Database) is a manually curated database which provides comprehensive experimentally validated information about the LMs mediating PPIs from all organisms on a single platform. About 2200 entries have been compiled by detailed manual curation of PubMed abstracts, of which about 1000 LM entries were being annotated for the first time, as compared with the Eukaryotic LM resource. The users can submit their query through a user-friendly search page and browse the data in the alphabetical order of the bait gene names and according to the domains interacting with the LM. LMPID is freely accessible at http://bicresources.jcbose. ac.in/ssaha4/lmpid and contains 1750 unique LM instances found within 1181 baits interacting with 552 prey proteins. In summary, LMPID is an attempt to enrich the existing repertoire of resources available for studying the LMs implicated in PPIs and may help in understanding the patterns of LMs binding to a specific domain and develop prediction model to identify novel LMs specific to a domain and further able to predict inhibitors/modulators of PPI of interest.

**Database URL:** http://bicresources.jcbose.ac.in/ssaha4/lmpid

## Introduction

Short contiguous stretches of amino acids, known as linear motifs (LMs), found within proteins, are known to mediate multiple protein–protein interactions (PPIs) in signaling and regulatory networks (1, 2). The LM instances approximately conform to a consensus sequence pattern and are often present in the disordered regions of proteins (3). The structural flexibility of these LM regions allows them to mediate transient and low affinity interactions with multiple interactors. Hence, the LMs may play an important role in shaping the spatio-temporal behavior of protein interaction networks (4, 5). Recently, LMs are being considered as novel targets for drug discovery against complex diseases and modulation of such interfaces using small chemicals is an emerging field of research (6–10). Examples of drugs developed using such strategy include Pfizer’s Selzentry (Maraviroc) used for treatment of HIV Infection, SARcode’s Lifitegrast ophthalmic solution and Roche's RG7112 (a potent and selective member of the Nutlin family of inhibitors of p53-MDM2 binding used in treatment of solid tumors) (10).

There are a few resources publicly available viz the eukaryotic linear motif (ELM) resource (11), Minimotif Miner (MnM) (12) and Scansite (13), which catalogue the experimental and predicted LMs. The ELM consortium was established in 2003 for providing a platform for storing, retrieving and analysing functional sequence motifs as well as for identification of new instances of the annotated motif patterns. Apart from the protein-binding motifs (LIG), the ELM database also contains motifs forming proteolytic cleavage sites (CLV), post-translational modification (PTM) sites (MOD) and sub-cellular targeting sites (TRG). In the 2014 release of ELM, docking and degradation motifs (DOC and DEG, respectively) have been removed from the LIG category and classified separately. MnM is a web-based motif-prediction tool that compares protein sequences with the motif instances in the MnM database, which includes motif involved in PPIs, PTMs and protein trafficking. The Scansite program uses a motif profile scoring algorithm to identify potential motifs within query protein sequences by comparing them with experimentally derived motif profile matrices. However, the data in MnM and Scansite can neither be browsed nor can be downloaded by the users. There is also a specialized database, PDZBase (14), containing PDZ domain-mediated interactions that have been manually extracted from literature.

Linear Motif mediated Protein Interaction Database (LMPID) is a manually curated database which provides comprehensive information about the LM instances mediating PPIs from all organisms. Unlike PDZBase, LMPID is not restricted to any single domain. PDZBase contains both domain-domain and domain-peptide interactions, whereas LMPID only includes domain-motif interactions. Again, ELM and MnM, compile a broad range of functional motifs, whereas, LMPID focuses only on motifs mediating PPIs, because these motifs may be targeted for modulation by small molecules. LMPID incorporates only experimentally validated motif instances, whereas ELM also includes the predicted ones. Furthermore, 1003 LM entries were being annotated for the first time, as compared with the ELM (‘LIG’, ‘DEG’ and ‘DOC’ classes). New fields giving information on critical residues and PTMs, like phosphorylation, affecting the PPI, disease associations and inhibitors (if any) have been introduced in LMPID which were not present in ELM. The overlapping ELM data (from ‘LIG’, ‘DEG’ and ‘DOC’ classes) have been extensively re-annotated with these new fields. Considerable amounts of missing information on the existing fields like secondary structure, interacting proteins and experimental evidences in support of the PPI, have been added. Overall, LMPID catalogues useful information on naturally occurring LM instances mediating PPIs that are experimentally validated and reported in literature, to provide reliable information about the key structural and functional aspects that may help in discovering novel modulators of PPIs involved in diseases.

## Data collection and annotation

About 8000 abstracts were downloaded from PubMed on 31 October 2014, using keywords like ‘motif’ and ‘interaction’ in the PubMed Advanced Search Builder. The ‘pubmed.mineR’ package (15) was used to shortlist the most relevant abstracts. In total, 1253 articles were studied to extract the details of any LM instance reported in it and the PPI mediated through this motif. The manually extracted information was used to meticulously annotate each entry of LMPID. Although a portion of the motifs collected by text mining were found to be overlapping with the motifs in the ELM resource, new fields with additional information were added, therefore enriching the information content of these ELM motifs.

## Data organization

### Database contents

LMPID contains information on the regular expression and sequence of the LM instance, the domain interacting with it, the experimental methods used to validate the instance and the PPI, the protein containing the motif (bait) and its interacting partner (prey). The motif table, indexed by the ‘Instance ID’, contains 2203 entries and the interaction table, linked to the motif entries through the ‘Interaction ID’, supplies information about the 2203 interactions mediated by each of the motif instances. Links have been provided to the respective UniProt, PubMed and PDB IDs. In total, LMPID contains 1750 unique LM instances mediating 2203 PPIs among 1181 baits and 552 prey proteins. A comparison of LMPID data with ELM data (from ‘LIG’, ‘DEG’ and ‘DOC’ categories) is shown in Table 1 and Supplementary Table S1, indicating that 655 unique additional instances were newly annotated in LMPID. A comparative statistics of different types of organisms and the different interacting domains represented in the LMPID data are given in Supplementary Tables S2 and S3, respectively.

**Table 1.** Comparison of LMPID with ELM (‘LIG’, ‘DOC’ and ‘DEG’ categories)

Data Source	Number of entries
ELM^a^	1698
LMPID	2203
Common in both	1200

^a^Contains predicted and false-positive instances also.

### Database schema

**Figure 1.**
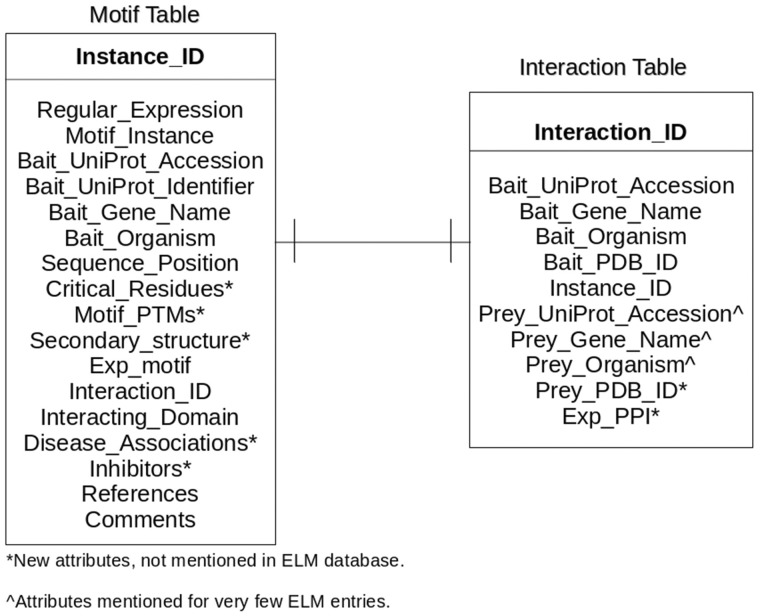
Entity-relationship diagram of LMPID. Asterisk (*) marked attributes present only in LMPID, whereas caret (^) marked attributes were substantially enriched as compared with ELM.

LMPID is a relational database comprising of two tables (i) the Motif table, for storing the motif instances with ‘Instance_ID’ as the primary key, and (ii) the Interaction table for storing the interactions mediated by the motif instances with ‘Interaction_ID’ as the primary identifier, as shown in Figure 1. Both the tables include the primary key of each other as foreign keys to enable connectivity. The Bait_UniProt_Accession, Bait_UniProt_Identifier and Bait_Gene_Name are the identifiers for the protein containing the LM instance. The new fields introduced in LMPID viz. Critical_Residues (positions of the motif critical for the interaction), Secondary_structure (secondary structure of the LM region) and others have been marked with an asterisk as shown in Figure 1. The fields of ELM with missing information like Prey_UniProt_Accession, Prey_Gene_Name and others have been marked with the “caret” (^) symbol.

## Implementation and data access

The LMPID database is implemented using Apache HTTP 2.2.15 web server and MySQL 5.1.69 database server. The web interface has been designed with PHP 5.3.3, HTML, JavaScript and CSS. It is freely accessible at *bicresources.jcbose.ac.in/ssaha4/lmpid*.

### Search and browse options

**Figure 2.**
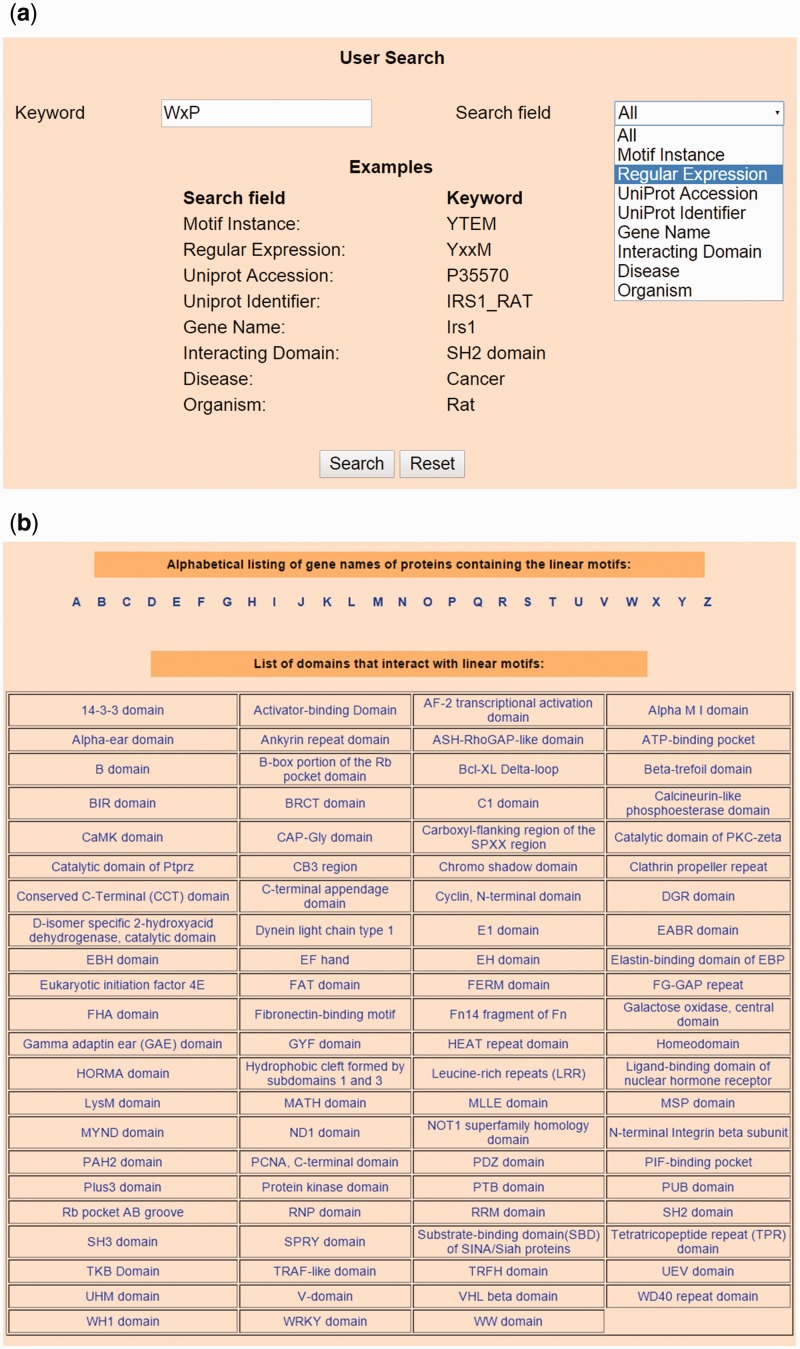
Snapshots of search and browse option of LMPID. (**a**) Search’ page of LMPID showing ‘WxP’ used as a keyword to be searched in the ‘Regular Expression’ field. (**b**) ‘Browse’ page of LMPID.

Users can submit specific search operations on the database using proper keywords through a user-friendly interface in the Home page, as shown in Figure 2a. The database can be queried on all fields (default option) or any one of the following fields—Motif Instance, Regular Expression, UniProt Accession, UniProt Identifier, Gene Name, Organism, domain interacting with the motif and diseases associated with the interaction. The ‘Browse’ page allows users to browse LMPID data in alphabetical order of gene names of bait proteins and according to the domains interacting with the LMs, as shown in Figure 2b. Users can download the LMPID data in csv or xml format from the ‘Download’ page.

### Information on the output page

**Figure 3.**
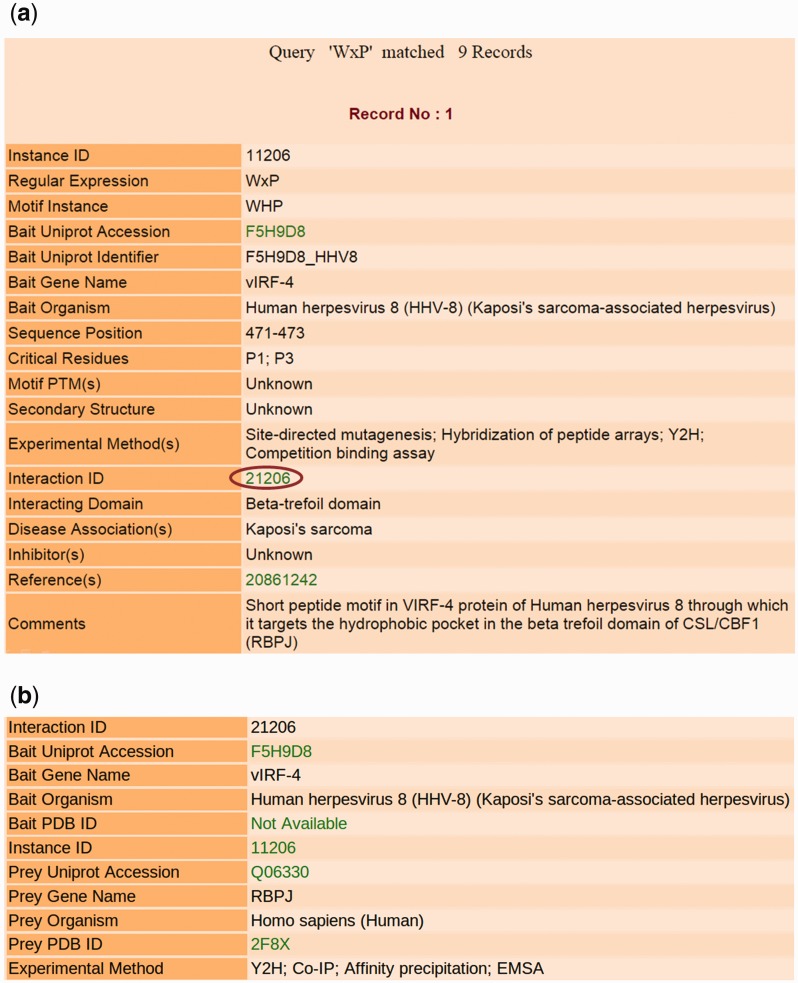
Output results of LMPID. (**a**) The main search result against the query ‘WxP’. (**b**) Page showing the details of the interacting bait and prey proteins by clicking on the hyperlink ‘Interaction ID’.

The output page contains comprehensive information about the motif entries retrieved by the user-submitted search or by browsing for gene names of proteins containing them or for domains binding them. Figure 3a shows a sample output page generated by querying the database using the keyword ‘WxP’ as the ‘Regular Expression’. The total number of records for each query search is provided on top of the output page. Each record contains the regular expression and sequence of the motif instance, the UniProt accession, UniProt identifier, gene and organism names of the bait protein containing this instance and sequence position of the bait protein where the instance is located. This motif class ‘WxP’ binding with ‘Beta-trefoil domain’ is not available in ELM (LIG, DEG and DOC classes) resource. The critical residues and the secondary structure of the motif instance as well as PTMs, if any, are also mentioned. Critical residues are those residues in the LM sequence whose mutation causes the PPI to be disrupted or the affinity of the interaction to be substantially decreased in magnitude. There is information about the domain that interacts with this motif, and the experimental procedures used to study the motif instance and its role in mediating the interaction. Effort has been made to provide information about any diseases associated with this interaction wherever possible, and a brief comment describing the interaction has been added to summarize all relevant information about the entry. All annotations have been extracted from the articles that report the experimental studies on the LM instance. The PubMed IDs of the reference articles providing information about the entry are hyperlinked to their respective PubMed entries. The UniProt accession is also linked to the corresponding UniProt page of the protein. Clicking on the ‘Interaction ID’ (as marked by a red circle) redirects to a page (shown in Figure 3b) containing information about the proteins interacting via the respective LM instance. This page mentions the organism names and PDB IDs (if any) of the bait protein containing the motif, as well as the prey protein interacting with it, and the experimental methods used to validate this interaction. The UniProt and PDB IDs are hyperlinked to their respective UniProt and PDB entries.

## Discussion and conclusion

LMPID describes the key structural and functional attributes of 2203 entries out of which 1750 were unique instances that mediate PPIs. Our aim is to provide a dedicated web-server with comprehensive experimentally validated information about the LMs mediating PPIs from all organisms, which shall be maintained for more than 5 years and updated at 6 months intervals. This is our first release, and we have a long term plan to update and maintain this database.

## Supplementary Data

Supplementary data are available at *Database* Online.

Supplementary Data

## References

[1] Diella F. Haslam N. Chica C. (2008) Understanding eukaryotic linear motifs and their role in cell signaling and regulation. Front Biosci., 13, 580–603. 18508681 10.2741/3175

[2] Neduva V. Russell R.B. (2006) Peptides mediating interaction networks: new leads at last. Curr Opin Biotechnol., 17, 465–471. 16962311 10.1016/j.copbio.2006.08.002

[3] Petsalaki E. Russell R.B. (2008) Peptide-mediated interactions in biological systems: new discoveries and applications. Curr. Opin. Biotechnol., 19, 344–350. 18602004 10.1016/j.copbio.2008.06.004

[4] Perkins J.R. Diboun I. Dessailly B.H. (2010) Transient protein–protein interactions: structural, functional, and network properties. Structure, 18, 1233–1243. 20947012 10.1016/j.str.2010.08.007

[5] Kim I. Lee H. Han S.K. Kim S. (2014) Linear motif-mediated interactions have contributed to the evolution of modularity in complex protein interaction networks. PLoS Comput. Biol., 10, e1003881. 25299147 10.1371/journal.pcbi.1003881 PMC4191887

[6] Roberts K.E. Cushing P.R. Boisguerin P. (2012). Computational Ddesign of a PDZ domain peptide inhibitor that rescues CFTR Aactivity. PLoS Comput. Biol., 8, e1002477. 22532795 10.1371/journal.pcbi.1002477 PMC3330111

[7] Groner B. Weber A. Mack L. (2012) Increasing the range of drug targets: interacting peptides provide leads for the development of oncoprotein inhibitors. Bioengineered, 3, 320–325. 22825353 10.4161/bioe.21272 PMC3489706

[8] Labbé C.M. Laconde G. Kuenemann M.A. (2013) iPPI-DB: a manually curated and interactive database of small non-peptide inhibitors of protein–protein interactions. Drug Discov. Today, 18, 19–20. 10.1016/j.drudis.2013.05.003 23688585

[9] Nero T.L. Morton C.J. Holien J.K. (2014) Oncogenic protein interfaces: small molecules, big challenges. Nat. Rev. Cancer, 14, 248–262. 24622521 10.1038/nrc3690

[10] Meier C. Cairns-Smith S. Schulze U. (2013) Can emerging drug classes improve R&D productivity? *Drug Discov**.* Today, 18, 13–14. 10.1016/j.drudis.2013.05.006 23702084

[11] Dinkel H. Van Roey K. Michael S. (2014) The eukaryotic linear motif resource ELM: 10 years and counting. Nucleic Acids Res., 42, D259–D266. 24214962 10.1093/nar/gkt1047 PMC3964949

[12] Mi T. Merlin J.C. Deverasetty S. (2012) Minimotif Miner 3.0: database expansion and significantly improved reduction of false-positive predictions from consensus sequences. Nucleic Acids Res., 40, D252–D260. 22146221 10.1093/nar/gkr1189 PMC3245078

[13] Obenauer J.C. Cantley L.C. Yaffe M.B. (2003) Scansite 2.0: Proteome-wide prediction of cell signaling interactions using short sequence motifs. Nucleic Acids Res., 31, 3635–3641. 12824383 10.1093/nar/gkg584 PMC168990

[14] Beuming T. Skrabanek L. Niv M.Y. (2005) PDZBase: a protein–protein interaction database for PDZ-domains. Bioinformatics, 21, 827–828. 15513994 10.1093/bioinformatics/bti098

[15] Sharma J. Ramachandran S. Rauf Shah Ab. (2014) Text mining of PubMed abstracts. R package version 1.0.

