# Supplementary material for: LMPID: A manually curated database of linear motifs mediating protein–protein interactions
Source: Database (Oxford). 2015 Mar 16;2015:bav014. doi: 10.1093/database/bav014 (PMC4360622; doi:10.1093/database/bav014)
Supplement: Supplementary Data [file supp_2015_bav014_index.html]

Supplementary Data 

# LMPID: A manually curated database of linear motifs mediating protein–protein interactions

## Supplementary Data

files

**Files in this Data Supplement:**

- Supplementary Data - zip file
