# Supplementary material for: LMPID: A manually curated database of linear motifs mediating protein–protein interactions
Source: Database (Oxford). 2015 Mar 16;2015:bav014. doi: 10.1093/database/bav014 (PMC4360622; doi:10.1093/database/bav014)
Supplement: Supplementary Data [file supp_bav014_suppl_data.zip › New Microsoft Office Word Document.docx]

**SUPPLEMENTARY INFORMATION**

**Table S1:** Comparison of LMPID with overlapping ELM data.

**Table S2:** Number of bait proteins from each organism.

**Table S3:** List of domains found to interact with the motif instances in LMPID.
