# Supplementary material for: LMPID: A manually curated database of linear motifs mediating protein–protein interactions
Source: Database (Oxford). 2015 Mar 16;2015:bav014. doi: 10.1093/database/bav014 (PMC4360622; doi:10.1093/database/bav014)
Supplement: Supplementary Data [file supp_bav014_suppl_data.zip › LMPID_supplementary_information.doc]

# LMPID: A manually curated database of linear motifs mediating protein-protein interactions.

Debasree Sarkar^1^, Tanmoy Jana^1^, Sudipto Saha^1*^

^1^ Bioinformatics Centre, Bose Institute, Kolkata, India.

**SUPPLEMENTARY INFORMATION**

**Table S1: Comparison of LMPID with overlapping ELM data.**

| **Data Source** | **Number of unique instances** | **Number of unique bait proteins** | **Number of unique prey proteins** |
| --- | --- | --- | --- |
| **ELM^#^** | 1095 | 760 | 354 |
| **LMPID** | 1750 | 1181 | 552 |

^#^ ELM entries overlapping with LMPID.

**Table S2: Number of bait proteins from each organism.**

| **Organism** | **Number of LMPID entries** |
| --- | --- |
| Homo sapiens (Human) | 1265 |
| Mus musculus (Mouse) | 306 |
| Viruses | 175 |
| Rattus norvegicus (Rat) | 131 |
| Yeast | 102 |
| Drosophila melanogaster (Fruit fly) | 77 |
| Arabidopsis thaliana (Mouse-ear cress) | 26 |
| Bacteria | 22 |
| Xenopus laevis (African clawed frog) | 16 |
| Bos taurus (Bovine) | 14 |
| Gallus gallus (Chicken) | 13 |
| Others | 56 |
| **Total** | 2203 |

**Table S3: List of domains found to interact with the motif instances in LMPID.**

| **Domain** | **Number of interacting instances** |
| --- | --- |
| PDZ domain | 273 |
| SH3 domain | 153 |
| WW domain | 152 |
| AF-2 transcriptional activation domain | 111 |
| 14-3-3 domain | 100 |
| EH domain | 84 |
| WD40 repeat domain | 52 |
| SH2 domain | 50 |
| PTB domain | 49 |
| EF hand | 37 |
| B domain | 34 |
| Hydrophobic cleft formed by subdomains 1 and 3 | 33 |
| WH1 domain | 33 |
| Ankyrin repeat domain | 32 |
| MATH domain | 29 |
| Calcineurin-like phosphoesterase domain | 26 |
| C-terminal appendage domain | 26 |
| D-isomer specific 2-hydroxyacid dehydrogenase, catalytic domain | 26 |
| MLLE domain | 26 |
| Ligand-binding domain of nuclear hormone receptor | 24 |
| Homeodomain | 22 |
| PCNA, C-terminal domain | 21 |
| Conserved C-Terminal (CCT) domain | 20 |
| Protein kinase domain | 20 |
| Substrate-binding domain(SBD) of SINA/Siah proteins | 19 |
| Cyclin, N-terminal domain | 18 |
| UEV domain | 18 |
| PIF-binding pocket | 17 |
| Tetratricopeptide repeat (TPR) domain | 14 |
| Eukaryotic initiation factor 4E | 13 |
| MYND domain | 13 |
| BRCT domain | 12 |
| Clathrin propeller repeat | 12 |
| Galactose oxidase, central domain | 12 |
| Gamma adaptin ear (GAE) domain | 12 |
| GYF domain | 11 |
| TKB Domain | 11 |
| Alpha M I domain | 10 |
| BIR domain | 10 |
| FHA domain | 10 |
| Alpha-ear domain | 9 |
| Beta-trefoil domain | 9 |
| TRAF-like domain | 9 |
| Dynein light chain type 1 | 8 |
| Elastin-binding domain of EBP | 8 |
| RRM domain | 8 |
| V-domain | 8 |
| Chromo shadow domain | 7 |
| EBH domain | 7 |
| PAH2 domain | 7 |
| CaMK domain | 6 |
| EABR domain | 6 |
| N-terminal Integrin beta subunit | 6 |
| Catalytic domain of Ptprz | 5 |
| FAT domain | 5 |
| HEAT repeat domain | 5 |
| MSP domain | 5 |
| SPRY domain | 5 |
| TRFH domain | 5 |
| VHL beta domain | 5 |
| WRKY domain | 5 |
| CB3 region | 4 |
| E1 domain | 4 |
| ND1 domain | 4 |
| ASH-RhoGAP-like domain | 3 |
| B-box portion of the Rb pocket domain | 3 |
| CAP-Gly domain | 3 |
| Leucine-rich repeats (LRR) | 3 |
| NOT1 superfamily homology domain | 3 |
| PUB domain | 3 |
| Rb pocket AB groove | 3 |
| UHM domain | 3 |
| Carboxyl-flanking region of the SPXX region | 2 |
| Catalytic domain of PKC-zeta | 2 |
| DGR domain | 2 |
| FERM domain | 2 |
| FG-GAP repeat | 2 |
| HORMA domain | 2 |
| RNP domain | 2 |
| Activator-binding Domain | 1 |
| ATP-binding pocket | 1 |
| Bcl-XL Delta-loop | 1 |
| C1 domain | 1 |
| Fibronectin-binding motif | 1 |
| Fn14 fragment of Fn | 1 |
| LysM domain | 1 |
| Plus3 domain | 1 |
